# Walnut (*Juglans regia* L.) Oligopeptides Alleviate Alcohol-Induced Acute Liver Injury through the Inhibition of Inflammation and Oxidative Stress in Rats

**DOI:** 10.3390/nu15092210

**Published:** 2023-05-06

**Authors:** Rui Liu, Yun-Tao Hao, Na Zhu, Xin-Ran Liu, Rui-Xue Mao, Jia-Wei Kang, Chao Hou, Ting Zhang, Yong Li

**Affiliations:** 1 Department of Nutrition and Food Hygiene, School of Public Health, Peking University, Beijing 100191, China; 2 Department of Nutrition and Food Hygiene, College of Public Health, Inner Mongolia Medical University, Hohhot 010059, China; 3 Department of Clinical Nutrition, Peking University People’s Hospital, Beijing 100044, China

**Keywords:** walnut oligopeptides, alcoholic liver disease, alcohol-metabolic enzymes, oxidative stress, inflammation

## Abstract

The study was aimed at investigating the effects of walnut oligopeptides (WOPs) on alcohol-induced acute liver injury and its underlying mechanisms. Male Sprague Dawley (SD) rats were randomly assigned to six groups: normal control, alcohol control, whey protein (440 mg/kg.bw), and three WOPs (220 mg/kg.bw, 440 mg/kg.bw, 880 mg/kg.bw) groups. After 30 days of gavage, ethanol with a volume fraction of 50%, administered at a dose of 7 g/kg.bw., caused acute liver injury. A righting reflex experiment and a blood ethanol concentration evaluation were then performed. Serum biochemical parameters, inflammatory cytokines, liver alcohol metabolism enzymes, oxidative stress biomarkers, liver nuclear factor-κB (NF-κB p65), and cytochrome P4502E1 expression were determined. The results revealed that the intervention of 440 mg/kg and 880 mg/kg WOPs could alleviate the degree of intoxication, decrease blood ethanol concentration, alleviate alcohol-induced hepatic steatosis, enhance the activity of hepatic ethanol metabolizing enzymes and antioxidant capacity, reduce lipid oxidation products and pro-inflammatory factor contents, and inhibit the expression of NF-κBp65 in the livers of rats. The outcomes of the study suggest that WOPs have beneficial effects on liver damage caused by acute ethanol binge drinking, with the high-dose WOPs (880 mg/kg.bw) exerting the most pronounced hepatoprotective effect.

## 1. Introduction

Alcohol is considered to be a serious factor in liver disease, and the subtypes of liver disease involved with ethanol include alcoholic hepatitis, steatosis, steatohepatitis, fibrosis, and cirrhosis, which are worldwide public health problems [1]. Acute alcoholic hepatitis and cirrhosis have a high mortality rate, with acute alcoholic hepatitis exhibiting a mortality rate of up to 50% [2,3]. Researchers have shown that the number of people with alcoholic liver disease (ALD) in China is growing at an amazing rate [4]. During the period of 2002–2013, the distribution of liver disease types at the Beijing 302 Hospital changed, with the proportion of ALD more than doubling [4]. In 2016, harmful alcohol use accounted for approximately 5.3% of total deaths globally, and 5.1% of all disability-adjusted life years (DALYs) [2,5]. Among males, an estimated 2.3 million deaths and 106.5 million DALYs were attributed to alcohol use in 2016 [2]. Health systems may soon be undergoing a large and growing demand for the treatment of alcoholic liver disease. However, there is no effective treatment for ALD except alcohol withdraw, and the current management options for alcoholic hepatitis, such as glucocorticoid therapy and enteral nutrition, remain inadequate [6]. Consequently, there is an urgent need to explore the potential role of new therapeutic approaches for ALD, particularly the hepatic consequences of acute ethanol binge drinking-induced hepatitis, in order to protect the liver at an early stage of disease development and slow down the progression of ALD.

Bioactive peptides are remarkable biomolecules with a wide spectrum of pharmacological functions, including anti-oxidant, antifungal, hypotensive, hypoglycemic, keratolytic, and sedative activities, which are encrypted in their sequences and released during digestion [7,8,9]. It has been reported that bioactive peptides have positive effects against alcohol induced liver damage [10,11,12,13]. Recently, peptides have attracted increasing interest because they have the potential to bridge the gap between small molecules and protein drugs by integrating the advantages of both [14]. Therefore, peptides extracted from natural foods, especially oligopeptides, may show promising effectiveness and safety in the prevention and mitigation of ALD.

Walnut (*Juglans regia* L.), as an important oil crop, is rich in protein, unsaturated fatty acids, calcium, iron, and many kinds of vitamins. It also contains rich nutritional and medicinal values, occupying an important position in both domestic and international food markets. With the development of modern medicine, a large number of pharmacological and clinical tests have found that walnuts have various therapeutic effects, such as enhancing immunity, improving memory, preventing cardiovascular diseases, and regulating abnormal lipid metabolism [15]. Yasir Uddin et al. found that walnut oil obtained from Madyan had similar properties to olive oil with high concentrations of oleic acid (41%) and linoleic acid (9%) [16]. Ullah, S. F. et al. found that walnut protein isolate showed excellent physicochemical and functional properties for use in food or industry product development [17]. However, previous studies mainly focused on walnut protein or oil, with little attention paid to walnut oligopeptides (WOPs). WOPs are mainly composed of oligopeptides with small molecular weight, but high activity, which are extracted and separated from walnut protein by enzymatic hydrolysis technology [18]. Animal studies have showed that WOPs exhibit a huge diversity of potential pharmacological functions, such as improving aging-related learning and preventing and memory decline, as well as anti-radiation and anti-fatigue properties [19,20]. Previous studies reported that the development of ethanol-caused liver damage is mainly related to the excessive oxidative stress and inflammation caused by ethanol metabolism, including hepatocyte steatosis, apoptosis, and fibrosis, etc. [21]. However, there are no reports regarding the effectiveness of WOPs against acute ethanol binge-caused liver injury. Hence, this study was implemented to investigate the potential effect of WOPs on the mitigation of acute alcohol binge drinking-induced liver injury and its possible mechanisms.

## 2. Materials and Methods

### 2.1. Materials and Reagents

WOPs were isolated from the common walnut by bienzymatic technology, purchased from Jilin Taigu Biological Engineering Co., Ltd. (Changchun, China). The analysis by high performance liquid chromatography found that the oligopeptides with a molecular weight less than 1000 in WOPs accounted for 86.5%, the total peptides less than 2000 accounted for 96.5%, and the contents of hydrolyzed amino acids and free amino acids were 53.15 g/100 g and 2.98 g/100 g, respectively. The safety evaluation experiments were conducted on WOPs, and the results showed that WOPs exhibited no obvious toxic effects, and its median lethal dose (LD50) was greater than 20 g/kg.bw, which was a non-toxic grade and safe for consumption [18,20].

The ethanol and tertbutanol used were of analytical grade and provided by Beijing Chemical Company (Beijing, China). Alcohol dehydrogenase (ADH) and acetaldehyde dehydrogenase (ALDH) kits were purchased from Beijing Multech Co., Ltd. (Beijing, China). Superoxide dismutase (SOD), reduced glutathione (GSH), catalase (CAT), malondialdehyde (MDA), reactive oxygen species (ROS), and protein carbonyls (PCO) were manufactured by Nanjing Jiancheng Bioengineering Institute (Nanjing, China). Alanine aminotransferase (ALT), aspartate aminotransferase (AST), and other serum biochemical detection reagents were obtained from Yingkexinchuang Science and Technology Ltd. (Beijing, China). The tumor necrosis factor α (TNF-α), diamine oxidase (DAO), D-lactate, lipopolysaccharide (LPS), interleukin (IL)-6, and interleukin (IL)-1β assay kits were purchased from Nanjing Jiancheng Bioengineering Institute (Nanjing, China). The primary antibodies against rabbit nuclear factor-κB p65 (NF-κB p65), CYP450 2E1 (rabbit), and inhibitor kappa Bα (IκBα) were purchased from ABclonal Technology Co., Ltd. (Wuhan, China).

### 2.2. Animals and WOPs Administration

Specific pathogen-free conditioned male Sprague Dawley (SD) rats weighing 180–220 g were purchased from the Department of Laboratory Animal Science, Peking University. The rats were housed in a barrier-level animal room with a temperature range of 22 ± 2 °C, a relative humidity of 50–60%, and a day/night alternation time of 12 h:12 h. The experiment was reviewed and approved by the Institutional Animal Care and Use Committee of Peking University, and all animals were treated according to the Principles of Laboratory Animal Care and the guidelines of the Peking University Animal Research Committee.

After one week of adaptive feeding, based on the body weight, SD rats were randomly assigned to six groups: normal control, alcohol control, whey protein (440 mg/kg.bw), and three WOPs groups (220 mg/kg.bw, 440 mg/kg.bw, 880 mg/kg.bw), with ten rats in per group. Rats in the three WOPs groups or whey protein group received WOPs or whey protein solutions (dissolved in distilled water at different doses) by intragastric gavage (i.g.), while rats in the normal and alcohol control groups were given distilled water in the same manner. Daily intragastric gavage intervention with a volume of 1 mL/100 g was performed for 30 days. During the experiment, the body weight of the rats was recorded once a week using electronic scales.

### 2.3. Blood Ethanol Concentration

After 30 days of intervention, rats in the alcohol control, whey protein, and three WOPs groups were administered with a dose of 4 g/kg of 50% volume ratio ethanol solution by intragastric gavage (i.g.), while rats in the normal control group were given an equal volume of saline. Blood samples were collected at 30, 60, 90, 120, and 180 min after administration of ethanol from tail bleeding, and the volume of blood collected was approximately 0.3 mL each time. A total of 0.2 mL was transferred to a headspace vial immediately after blood collection, and 0.2 mL of internal standard (tert-butanol solution 0.6 mg/mL) was added and sealed immediately. The blood concentrations in the rats were then examined by headspace gas chromatography (GC). GC conditions: inlet: 200 °C, detector: FID 220 °C, carrier gas: nitrogen (99.999%), flow rate: 10 mL/min, column: DB-WAX (30 m × 0.53 mm), chromatographic conditions: start at 50 °C, hold for 2 min, ramp up to 60 °C at 10 °C/min, ramp up to 100 °C at 20 °C/min, ramp up to 200 °C at 40 °C/min, increase to 200 °C hold for 2 min.

### 2.4. Righting Reflex Experiment

The experiment was conducted to observe the drunkenness and sobriety of the rats with the rollover reflex. After the blood ethanol concentration test, the rats were placed back in their feeding cages and rested for one week. Then, the rats in the alcohol control, whey protein, and three WOPs groups received a 50% volume fraction of ethanol with a dose of 7 g/kg.bw. by intragastric instillation (i.g.), and the normal control group received an equal volume of saline (17.5 mL/kg.bw). Immediately after the administration of alcohol to the rats, the rats were placed in animal cages with their backs facing downward, and the reflexes were considered lost (i.e., intoxicated) if the rats could not turn over on their own within 30 s. When the rats could turn over on their own, the reflexes were considered restored (i.e., sober), and the number and duration of rats in each group that lost their righting reflex were recorded. The endpoint of the test was set at 16 h after alcoholic gavage, with fasting, but no restriction of water during the observation period.

### 2.5. Assay for Serum Biochemical Markers

After the righting reflex experiment, blood samples were obtained via the femoral artery, and serum was prepared by centrifugation at 3000 rpm for 15 min at 4 °C. The liver tissues were immediately isolated for further analysis. Then, serum ALT, AST, TC, TG, LDL-C, HDL-C, ALP, TP, ALB, GLB, CR, and BUN concentrations were detected by an automatic biochemical analyzer (Olympus Corporation, Tokyo, Japan), according to kit instructions.

### 2.6. Histopathological Examination

The liver tissues (*n* = 6 per group) were fixed with 4% paraformaldehyde solution, and then paraffin embedded after gradient dehydration using ethanol. Hematoxylin/eosin (HE) was used for staining. The slides were observed with an Olympus IX70 inverted microscope (Olympus, Tokyo, Japan) at a magnification of ×100 to ×400. Liver steatosis was assessed by observing the fat accumulation in the liver, which was classified into five grades. The scoring criteria was: grade 0 refers to lipid droplets in the hepatocytes as scattered, rare, and normal; grades I, II, and III refer to hepatocytes with lipid droplets no more than 1/4, 1/2, and 3/4 of the entire picture, respectively; the most severe grade IV refers to lipid droplets affecting almost the entire tissue of the liver.

### 2.7. Detection for Oxidative Stress Biomarkers

The contents of liver SOD, GSH-Px, CAT, GSH, ROS, PCO, and MDA were detected according to the kits instructions, as listed in Section 2.1. In brief, SOD was detected by the WST-1 method, GSH-Px by the colorimetric method, CAT by the ammonium molybdate spectrophotometric method, GSH by the microplate method, ROS by the chemiluminescence method, PCO by the UV colorimetric method, and MDA by the TBA method.

### 2.8. Enzyme-Linked Immunosorbent Assay

The levels of ADH and ALDH in the liver, and TNF-α, IL-6, IL-1β, LPS, DAO, and D-lactate in the serum were measured by enzyme-linked immunosorbent assay (ELISA), according to the operating procedures of the kit instructions, as listed in Section 2.1.

### 2.9. Western Blot Analysis

The expression of CYP2E1, NF-κB p65, and IκBα in the liver was determined by Western blot, analysis as previously reported, with some optimization modifications [18,21]. Briefly, the extraction and concentration of the total protein in the liver was determined by using RIPA lysis buffer and the BCA method. Then, 20 ug of each protein sample was electrophoresed through sodium dodecyl sulfate-polyacrylamide gel at a concentration of 12% and electro transferred to a polyvinylidene difluoride membrane (Millipore, Billerica, MA, USA). After one hour, the membranes were completely immersed in 3% (*m*/*v*) BSA-TBST and incubated for 0.5 h at room temperature. Next, incubation was conducted with primary antibodies against NF-κB at 1:200, IκBαa at 1:1000, CYP2E1 at 1:1000, and β-Actin at 1:200. After washing three times with TBST, the membrane was incubated with secondary antibody goat anti-rabbit IgG (1:5000) for 40 min at room temperature. The protein bands were examined visually by ECL reaction and film exposure, then quantified and handled with Total Lab Quant V11.5 (Newcastle upon Tyne, UK).

### 2.10. Statistics

Data are presented as mean ± SEM for bar and line graphs. A one-way analysis of variance (ANOVA) test, with least significant difference (LSD) methods, was used to determine the differences between groups when the data were homogeneous, or the nonparametric test (Kruskal–Wallis) was adopted if the equal variances were not assumed. Statistical analyses were conducted using SPSS software version 24 (SPSS Inc., Chicago, IL, USA). The rate of LORR was analyzed by Fisher’s exact probability test. A value of *p* < 0.05 was considered to be a statistically significant difference.

## 3. Results

### 3.1. Effect of WOPs on Body Weight and Organ Index

**Figure 1:**
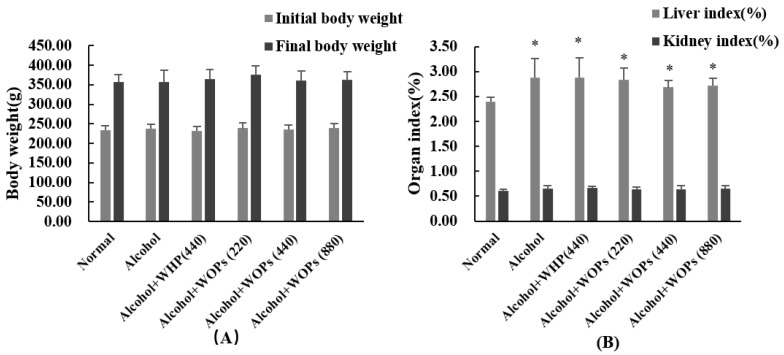
Effects of WOPs on body weight (**A**) and organ index (**B**) in rats. * *p* < 0.05 versus normal control group. WHP, whey protein group; WOPs, walnut oligopeptides. The organ index was calculated as a ratio (%) of the organ weight (g) to body weight (g).

After 30 days of daily oral administration of WOPs, there was no significant change in the final body weight among all groups observed (*p* > 0.05) (Figure 1A), and no rat died during the experiment. The liver index of the normal control group was lower than that of the five alcohol-treated groups (*p* < 0.05), which may be related to the acute inflammation of the liver induced by binge drinking, resulting in diffuse enlargement of the liver, but there was no significant difference regarding the kidney index (*p* > 0.05) (Figure 1B).

### 3.2. The Loss of Righting Reflex (LORR) Assay

As shown in Table 1, the ingestion of ethanol at 7 g/kg body weight after the WOPs administration could induce the incidence of LORR in 80% of the alcohol control rats after 36 min. Compared with alcohol control group, 440 mg/kg and 880 mg/kg of WOPs intervention greatly inhibited the incidence of LORR and prolonged the latency of LORR, while the incidence of LORR was reduced by 50% compared to that in the alcohol control group and the whey protein group, and the latency time of LORR was shortened by 78.58 and 92.58 min compared to that of the alcohol control group, respectively (*p* < 0.05, *p* < 0.01). In addition, the LORR duration of WOPs was markedly shorter in the 440 mg/kg and 880 mg/kg groups than in the whey protein group, with reductions of 262.7 and 249.37 min, respectively (*p* < 0.05).

### 3.3. Effect of WOPs on Blood Ethanol Concentration of Mice

**Figure 2:**
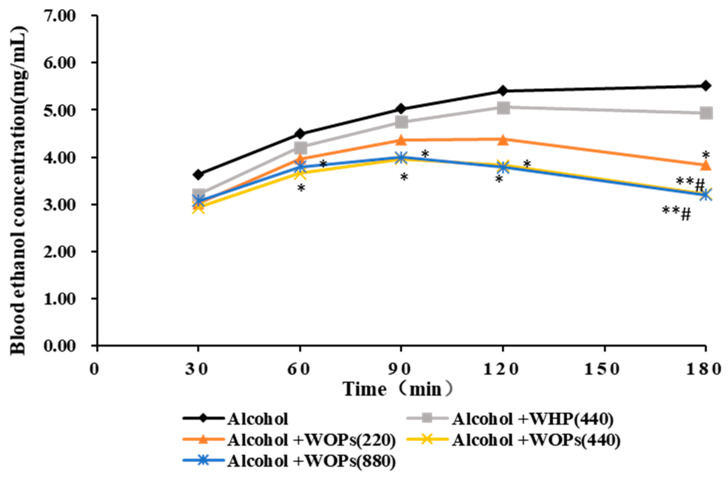
Effect of WOPs on blood ethanol concentration of rats after ethanol administration. * *p* < 0.05, ** *p* < 0.01, versus alcohol group; # *p* < 0.05 versus whey protein group. WHP, whey protein; WOPs, walnut oligopeptides.

Blood ethanol concentration (BEC) was measured at 30, 60, 90, 120, and 180 min after ethanol administration. As shown in Figure 2, the BEC of the alcohol control group and whey protein group increased over time. Compared to the alcohol control group, BEC was significantly lower in the medium- and high-dose WOPs groups at all time points, except for 30 min, with the medium-dose WOPs group showing reductions of 0.84, 1.06, 1.58, and 2.29 mg/mL at 60, 90, 120, and 180 min, respectively; the high-dose WOPs group showed reductions of 0.7, 1.03, 1.63, and 2.41 mg/mL, respectively (*p* < 0.05 or *p* < 0.01). In addition, the BEC of the low-dose WOPs group was dramatically lower than that of the alcohol control group by 1.67 mg/mL at 180 min (*p* < 0.05). Furthermore, the BEC of the medium- and high-dose WOPs groups was remarkably lower than that of the whey protein group at 180 min, with a reduction of 1.72 mg/mL in the medium-dose WOPs group and 1.74 mg/mL in the high-dose WOPs group. (*p* < 0.05).

### 3.4. Effect of WOPs on Serum Biochemical Parameters

As shown in Table 2, serum ALT and AST levels were remarkably elevated in the alcohol group when compared with the normal control group (*p* < 0.01). The levels of ALT and AST in three WOPs groups were remarkably lower than those in the alcohol group (*p* < 0.01), and there was no significant difference between the WOPs intervention groups and normal control group (*p* > 0.05).

Compared with the normal control group, the triglyceride (TG) levels in the alcohol control group were greatly elevated (*p* < 0.01). After 30 days of pretreatment with WOPs, in comparison with the alcohol control group, the TC levels in the medium-dose WOPs group was increased, while the contents of very low-density lipoprotein (VLDL) were significantly reduced in the high-dose WOPs group (*p* < 0.05 for TG, VLDL, *p* < 0.01 for LDL-C). However, the contents of high-density lipoprotein cholesterol (HDL-C) and total cholesterol (TC) did not differ significantly among all groups (*p* > 0.05).

The contents of serum total proteins (TP), albumin (ALB), globulin (GLB), and the ratio of albumin to globulin (A:G) in the alcohol control group were remarkably lower than those in the normal control group (*p* < 0.05 for ALB, *p* < 0.01 for TP, GLB, and A: G). The TP, ALB, GLB, and A:G levels were increased in three WOPs intervention groups, in which TP, GLB, and A:G values in the medium- and high-dose WOPs groups were dramatically different from those in the alcohol control group (*p* < 0.01).

The contents of alkaline phosphatase (ALP) were significantly higher, and creatinine (CR) and total bilirubin (TBil) were lower in the alcohol control group compared with those of the normal control group (*p* < 0.05 for CR, *p* < 0.01 for ALP and TBil). Compared with the alcohol control group, ALP activities in three WOPs intervention groups were remarkably lower (*p* < 0.01), but CR, TBil, and blood urea nitrogen (BUN) contents were not significantly different (*p* > 0.05).

### 3.5. Pathological Observations and Steatosis Grade

**Figure 3:**
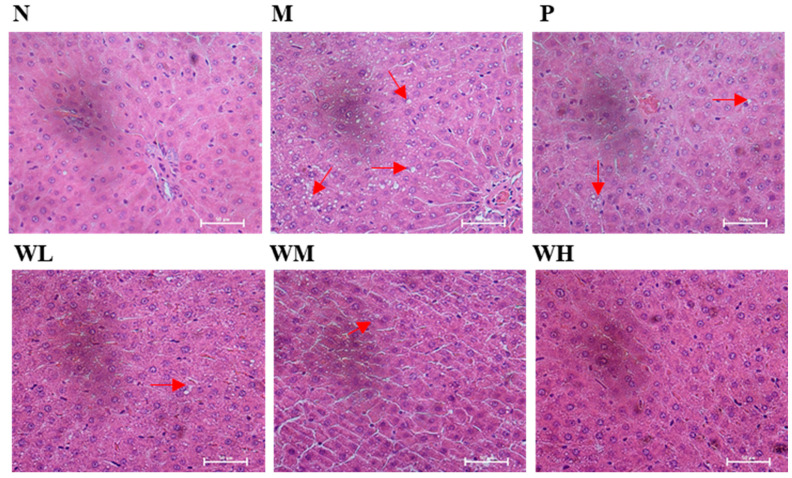
Histopathological assay of liver tissue sections with H&E. N, normal group; M, alcohol group; P, alcohol + whey protein group; WL, alcohol + WOPs (220) group; WM, alcohol + WOPs (440) group; WH, alcohol + WOPs (880) group. Representative photomicrographs with H&E staining reveal histopathological changes in the liver (scale bar, 50 μm), and prominent changes indicated by red arrows.

Histopathological studies revealed that treatment with high doses of ethanol induced severe hepatic steatosis, with obvious fat droplets in more than 75% of hepatocytes (Figure 3M) compared to rates found the normal control group (Figure 3N). Conversely, these pathological changes were attenuated in the WOPs groups (Figure 3WL–WH), especially in the high-dose WOPs groups, in which only minor hepatic steatosis (Figure 3WH) was observed before alcohol exposure. As shown in Table 3, compared with the normal group, liver steatosis in the alcohol group was significantly aggravated (*p* < 0.05). There was no significant difference between the WOPs groups and the alcohol group (*p* > 0.05), but there was no grade IV observed in the WOPs intervention groups, which indicated that pretreatment of WOPs may have potential effect of alleviating alcohol-induced liver steatosis.

### 3.6. Effect of WOPs on Alcohol-Metabolic Enzymes in Rat Liver

**Figure 4:**
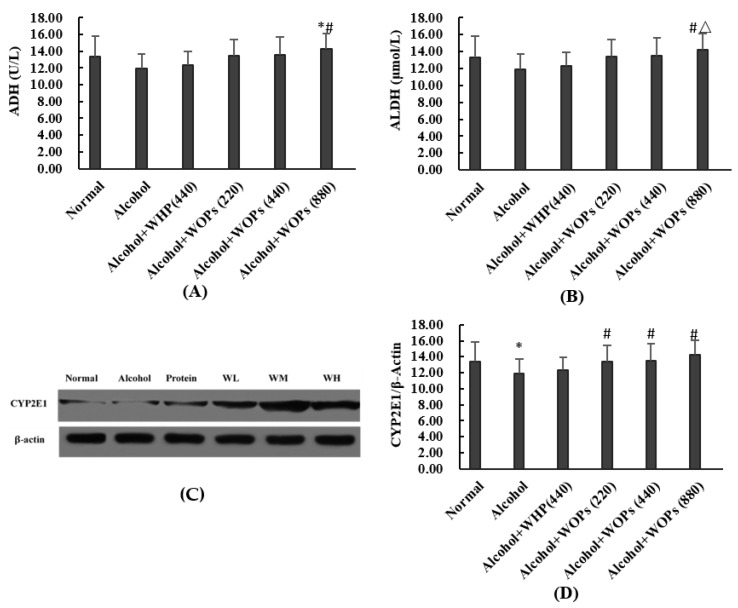
Effect of WOPs on activities of ADH (**A**), ALDH (**B**) and CYP2E1 (**C**,**D**) in livers of rats. * *p* < 0.05, versus normal control group; # *p* < 0.05 versus alcohol group; Δ *p* < 0.05 versus whey protein group. Protein, alcohol + whey protein group; WL, alcohol + WOPs (220) group; WM, alcohol + WOPs (440) group; WH, alcohol + WOPs (880) group. WHP, whey protein; WOPs, walnut oligopeptides.

Alcohol dehydrogenase (ADH), aldehyde dehydrogenase (ALDH) and cytochrome P450 play very important roles in the catabolism of alcohol in the liver. In this study, ADH activity was notably higher in the WOPs high-dose group (intervention dose of 880 mg/kg.bw) than in the normal control group and the alcohol control group (*p* < 0.05) (Figure 4A). ALDH activity was considerably higher in the WOPs high-dose group than in the alcohol group and the whey protein control group (*p* < 0.05) (Figure 4B). CYP2E1 protein expression was notably increased in the WOPs medium- and high-dose group compared to that in the alcohol group and the whey protein group (*p* < 0.05 or *p* < 0.01) (Figure 4D).

### 3.7. Effect of WOPs on Oxidative Stress Products in Liver of Rats

**Figure 5:**
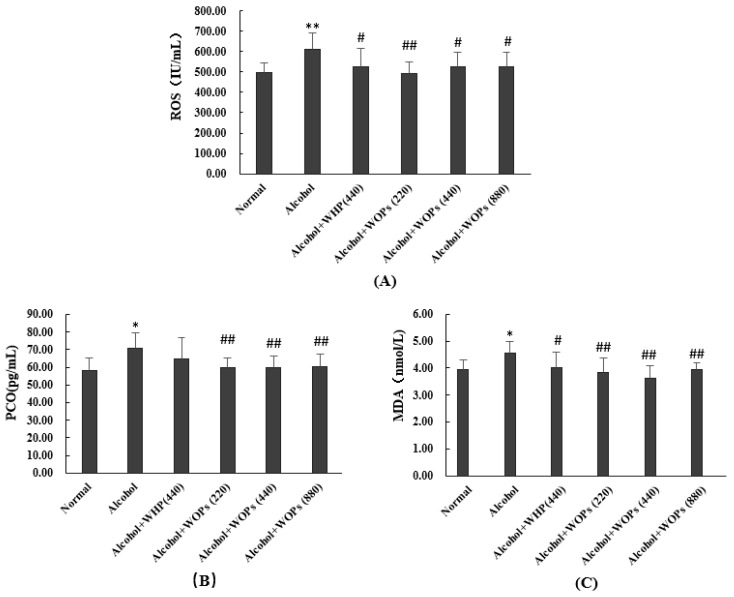
Effect of WOPs on activities of ROS (**A**), PCO (**B**), and MDA (**C**) in the livers of rats. * *p* < 0.05, ** *p* < 0.01, versus normal control group; # *p* <0.05, ## *p* <0.05 versus alcohol group. WHP, whey protein; WOPs, walnut oligopeptides.

As shown in Figure 5, the levels of reactive oxygen species (ROS), protein carbonyls (PCO), and malondialdehyde (MDA) were notably higher in the alcohol group compared with the levels in normal control group (*p* < 0.05 or *p* < 0.01), suggesting that the one-time high-dose alcohol intake induced the excessive oxidative stress response and caused the accumulation of a large number of oxidative stress products in vivo. In contrast, ROS, PCO, and MDA contents were remarkably lower in the three WOPs intervention groups compared with the alcohol group (*p* < 0.05 or *p* < 0.01), indicating that WOPs could inhibit the binge drinking-induced excessive oxidative stress in rats.

### 3.8. Effect of WOPs on Anti-Oxidation Capacity in Livers of Rats

**Figure 6:**
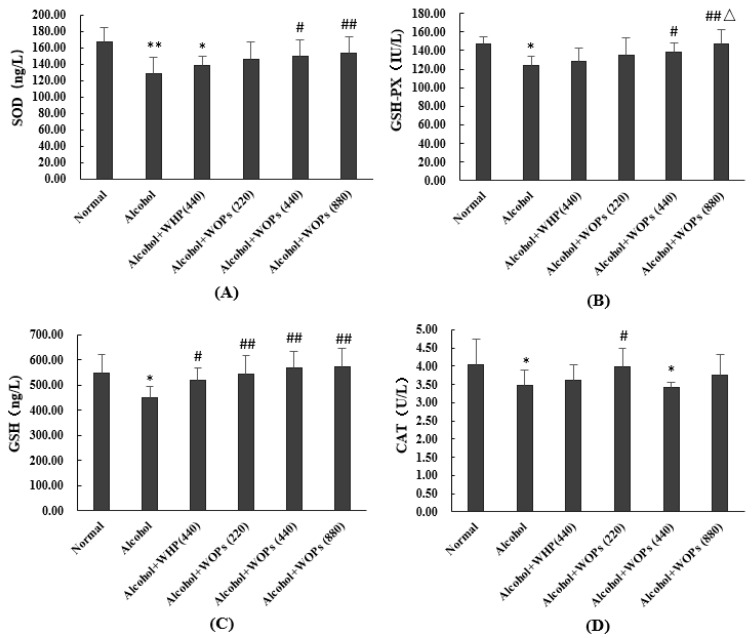
Effect of WOPs on the levels of SOD (**A**), GSH-Px (**B**), GSH (**C**), and CAT (**D**) in the liver of rats. * *p* < 0.05, ** *p* < 0.01, versus normal control group; # *p* < 0.05, ## *p* < 0.05 versus alcohol control group; Δ *p* < 0.05 versus whey protein group. WHP, whey protein; WOPs, walnut oligopeptides.

As shown in Figure 6, the levels of SOD, GSH-Px, CAT, and GSH were considerably lower in the alcohol groups compared with the normal control group (*p* < 0.05 or *p* < 0.01), indicating that binge drinking caused a large depletion of antioxidant substances in vivo. On the other hand, SOD (Figure 6A) and GSH-Px (Figure 6B) activities were notably elevated in the WOPs medium- and high-dose groups than in the alcohol group, and GSH-Px activity was remarkably increased in the WOPs high-dose group than in the whey protein group (*p* < 0.05 or *p* < 0.01). GSH contents were significantly higher in all three WOPs intervention groups when compared with that in the alcohol group (*p* < 0.01) (Figure 6C). CAT contents were significantly higher in the WOPs low-dose group than in the alcohol group (*p* < 0.05) (Figure 6D).

### 3.9. Effect of WOPs on Serum Pro-Inflammatory Cytokine Levels in Rats

**Figure 7:**
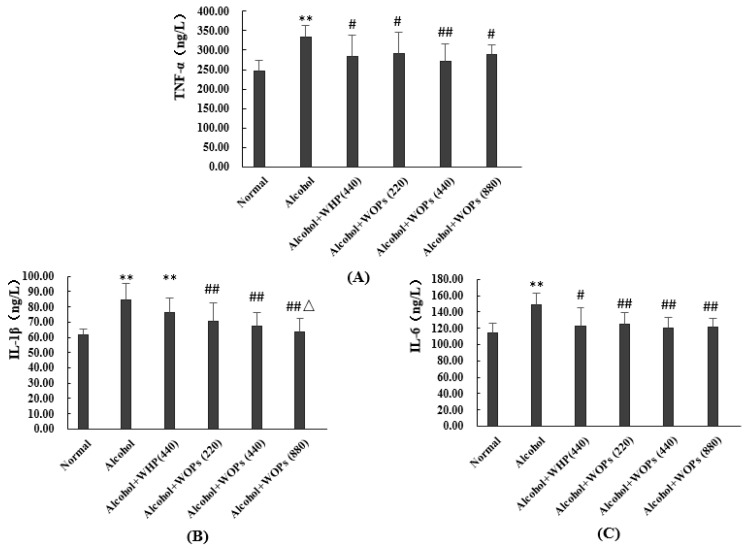
Effect of WOPs on the levels of serum TNF-α (**A**), IL-1β (**B**), and IL-6 (**C**) in rats. ** *p* < 0.01, versus normal control group; # *p* < 0.05, ## *p* < 0.05 versus alcohol control group; Δ *p* < 0.05 versus whey protein group. WHP, whey protein; WOPs, walnut oligopeptides.

Binge drinking can cause excessive inflammatory responses and produce large amounts of inflammatory cytokines. In this study, as shown in Figure 7, the levels of tumor necrosis factor-α (TNF-α), interleukin-6 (IL-6), and interleukin-1β (IL-1β) were significantly higher in the alcohol group than in the normal control group (*p* < 0.01). By contrast, the levels of TNF-α, IL-6, and IL-1β were remarkably lower in all three WOPs groups than in the alcohol group (*p* < 0.05 or *p* < 0.01), suggesting that WOPs could strongly suppress the accumulation of alcohol-induced inflammatory factors.

### 3.10. Effect of WOPs on Intestinal Permeability in Rats

**Figure 8:**
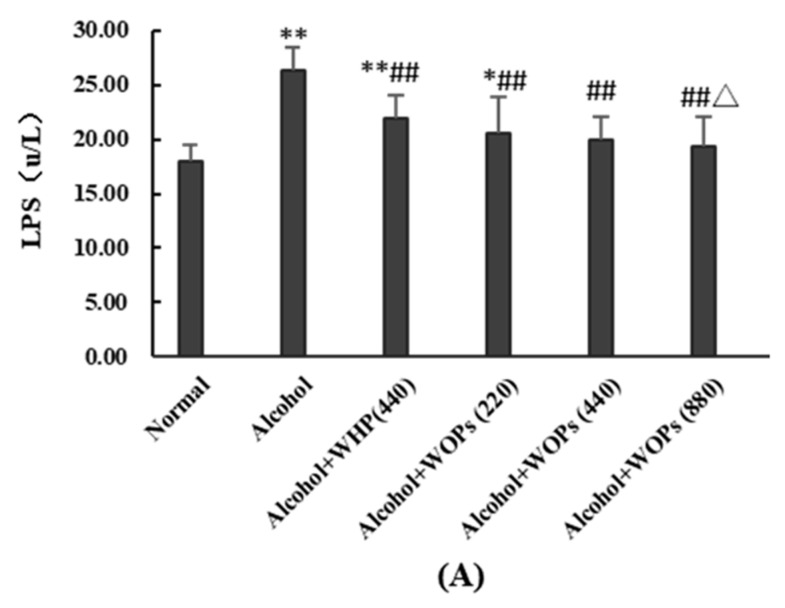

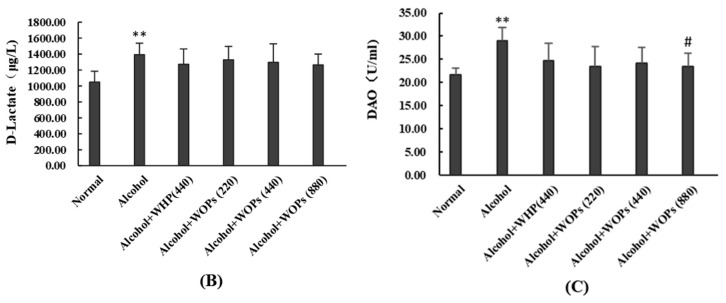
Effect of WOPs on the levels of serum LPS (**A**), D-lactate (**B**), and DAO (**C**) in rats. * *p* < 0.05, ** *p* < 0.01, versus normal control group; # *p* < 0.05, ## *p* < 0.05 versus alcohol control group; Δ *p* < 0.05 versus whey protein group. WHP, whey protein; WOPs, walnut oligopeptides.

As shown in Figure 8, the contents of serum lipopolysaccharide (LPS), diamine oxidase (DAO), and D-lactate were significantly increased in the alcohol group than in the normal control group (*p* < 0.01). Conversely, LPS levels were noticeably lower in all three groups of WOPs (*p* < 0.01) (Figure 8A), and DAO levels were significantly decreased in the WOPs high-dose group compared to the alcohol group (*p* < 0.05) (Figure 8C). Moreover, LPS levels were notably decreased in the WOPs high-dose group than in the whey protein group (*p* < 0.05) (Figure 8A).

### 3.11. Effect of WOPs on the Expression of Hepatic NF-κB p65 and IκBα in Rats

**Figure 9:**
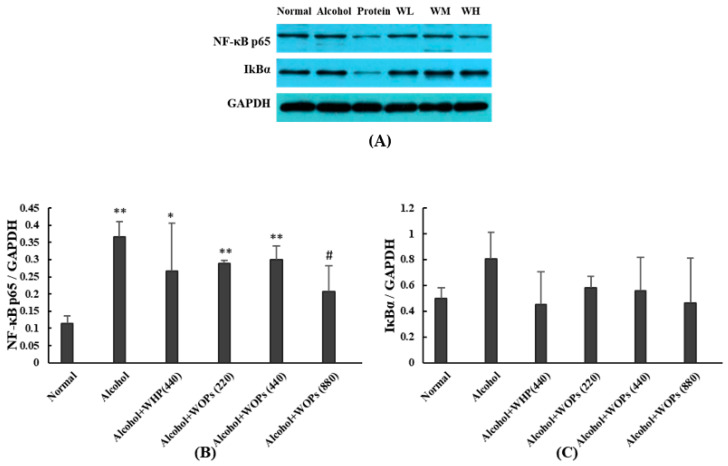
Effect of WOPs on the expression of hepatic NF-κB p65 (**B**) and IκBα (**C**) in rats (*n* = 3 for each group). (**A**), the Western blot strip in all groups. * *p* < 0.05, ** *p* < 0.01, versus normal control group; # *p* < 0.05, versus alcohol control group. WHP, whey protein; WOPs, walnut oligopeptides.

Hepatic nuclear factor-κB (NF-κB p65) and Inhibitor κBα (IκBα) binding is in an inactive state and under normal conditions, when external stimuli activate and phosphorylate IκBα, NF-κB p65 is then dissociated and phosphorylated to the nucleus. In this study, NF-κB p65 expression levels were significantly elevated in the alcohol, whey protein, and WOPs low- and medium-dose groups compared to those in the normal control group (*p* < 0.05 or *p* < 0.01) (Figure 9B). In contrast, NF-κB p65 expression levels were notably reduced in the WOPs high-dose group compared to those in the alcohol group (*p* < 0.05) (Figure 9B). However, there was no significant difference in the IκBα expression observed between groups (*p* > 0.05) (Figure 9C).

## 4. Discussion

Alcohol is the most serious risk factor of liver disease, which can induce liver function damage, resulting in hepatic steatosis, inflammatory response, and hepatocyte apoptosis [22]. Previous studies have mainly focused on ALD induced by long-term alcohol intake, and the effects of acute alcohol intoxication on ALD have been underappreciated. In this study, based on the previous studies and pre-experiments, gavage was administered at a dose of 7 g/kg.bw ethanol to cause an acute alcoholic liver injury in rats [23,24,25]. In order to exclude false positive results caused by simply increasing the protein intake, a whey protein control group was set up, in addition to the distilled water control group. Whey protein is rich in nutritional value, contains a complete range of essential amino acids in adequate amounts and in appropriate proportions, and has various physiological functions, including antioxidation and immunomodulation [26]. In this study, WOPs were administered by intragastric gavage (i.g.), dissolved in distilled water at different doses. It is possible that part of the walnut oligopeptide dose could be digested by intestinal peptidases to free amino acids, with subsequent adsorption into bloodstream. WOPs are rich in arginine, phenylalanine (Phe), tyrosine (Tyr), leucine (Leu), threonine (Thr), alanine (Ala), and glutamic acid (Glu), which have important physiological functions and are important nutrients in living organisms [19]. Therefore, the nutritional supplementation effect and various physiological activities may be mechanisms by which WOPs exert their hepatoprotective effects.

The righting reflex experiment reflects the degree of intoxication in rats [27]. Loss of the right reflex measurement indicates that the rat has entered the lethargic phase, implying a deeper level of acute alcohol intoxication and indicating the optimal time point to test the effectiveness of pharmacological interventions on intoxication [28]. The results of the present study showed that WOPs were able to reduce the disappearance rate of the righting reflex, with 80% of rats in the alcohol group falling into a period of lethargy and only 30% in the medium- and high-dose groups of WOPs. Moreover, the WOPs intervention significantly delayed and shortened the duration of intoxication compared with that of the alcohol and whey protein groups, illustrating that the WOPs intervention could significantly reduce the rate of intoxication and alleviate the degree of intoxication in rats. Blood ethanol concentration is closely related to intoxication, and detection of changes in blood ethanol concentration can be used to examine the effect of WOPs on ethanol metabolism [29]. The results of this study suggested that the blood ethanol concentrations in the WOPs medium- and high-dose groups were significantly reduced compared to those in the alcohol group, and the effect was better than that in the whey protein group, indicating that the WOPs intervention could effectively reduce blood ethanol concentration, exerting a significant preventive effect on acute alcohol intoxication.

There are more than 20 aminotransferases contained in vivo which enter the blood when hepatocytes are destroyed, and the damage to hepatocytes can be reflected by measuring the activity of aminotransferases [30]. Elevated serum ALT and AST are most sensitive in acute hepatocellular injury caused by various drugs, alcohol, harmful chemicals, or viral hepatitis [31,32]. The results of this study showed that serum ALT and AST levels were significantly higher in the alcohol group compared to the normal control group, indicating that acute liver function injury occurred in the alcohol group. ALT and AST levels in the WOPs intervention groups were significantly reduced, indicating that WOPs had a protective effect on alcohol-induced acute liver injury in rats. The total proteins mainly reflect the reserve capacity of the liver, and ALB mainly reflects the synthesis capacity of the liver [23]. When the liver function is damaged, and the synthesis function is impaired, the albumin content will be significantly reduced, causing a decrease in the total protein content. The results showed that serum TP and GLB levels were significantly reduced in the alcohol, whey protein, and three WOPs groups compared to those in the normal control group, which indicated that alcohol intake caused liver function damage and affected the protein synthesis function of the liver, which may be related to acute hepatitis caused by acute alcohol intoxication. However, the levels of TP, GLB, and the ratio of albumin to globulin were significantly increased in the medium- and high-dose groups of WOPs compared with the alcohol group, indicating that the WOPs intervention could have an improving effect on hepatic protein synthesis.

Abnormal lipid metabolism is very common in patients with alcoholic liver injury, and hepatocellular steatosis is an early response of the body to alcohol intake [33]. The metabolism of ethanol in the liver increases the level of the reduced form of nicotinamide-adenine dinucleotide (NADH), which increases the NADH/NAD ratio, causes changes in the redox state of the liver, inhibits glycogen synthesis, and promotes the elevation of lipid synthase activity, resulting in increased lipid synthesis [34,35]. In addition, ethanol metabolism also increases the activity of the sterol regulatory element binding proteins and inhibits the transfer of fatty acids into the mitochondria for oxidation and degradation under the regulation of malonyl-CoA, resulting in the deposition of lipids in the liver and leading to alterations in lipid levels [36]. The liver pathology results showed significant hepatocyte steatosis and many lipid droplet vacuoles in the alcohol group, while hepatic steatosis was significantly reduced in all groups of WOPs compared with the alcohol group, and the hepatocytes were clearly defined and regularly arranged. Moreover, the serum TG level was significantly higher in the alcohol group compared with the normal control group, while the TG level in the WOPs high-dose group was significantly reduced compared to that in the alcohol and whey protein groups, indicating that the WOPs intervention could alleviate hepatocyte damage, maintain the normal structure of the liver, and improve the alcohol-induced disorders of lipid metabolism.

More than 90% of the alcohol consumed by the body is metabolized in the liver, where it is first oxidized to acetaldehyde through the hepatocyte ADH, the microsomal ethanol oxidizing system (MEOS), and the peroxidase system [37]. Acetaldehyde is then oxidized by ALDH to acetic acid, which is converted to acetyl coenzyme A to enter the tricarboxylic acid cycle, with the end products being carbon dioxide and water. MEOS function is dependent on CYP450 [38,39]. CYP2E1 is a key member of the CYP450 family that mediates the effects of liver damage induced by a variety of compounds, and there is a significant correlation between its expression level and hepatotoxicity [40]. CYP2E1 was found to be closely associated with the development of fatty liver disease, and liver steatosis due to alcohol intake was more severe in normal mice compared to CYP2E1 knockout mice [41]. Ethanol and its metabolites, such as acetaldehyde and acetic acid, have direct hepatotoxic effects and can directly or indirectly cause inflammatory responses, oxidative stress, and severe hepatic hypoxia [42]. In this study, the results showed that the liver ADH and ALDH contents of rats were significantly elevated in the WOPs high-dose group, and CYP2E1 expression was significantly upregulated in the middle- and high-dose groups of WOPs, indicating that the intervention of WOPs increased the activity of ethanol-related metabolic enzymes and promoted the catabolism and metabolism of ethanol, mitigating liver damage caused by alcohol and its harmful metabolites.

Excessive oxidative stress induced by alcohol is considered to be a critical mechanism of ALD [13,43]. Ethanol intake activates the microsomal ethanol oxidase system in the endoplasmic reticulum; meanwhile, there is a sizeable production of free radicals in this metabolic pathway, particularly an accumulation of ROS, leading to a series of damage to the hepatocytes and even inducing apoptosis, of which lipid peroxidation is one manifestation [44,45]. PCO is formed by the reaction of ROS with amino acid residues in proteins, and elevated levels of PCO have been observed in patients with alcoholic liver injury [43]. MDA is a strong cytotoxic end product of lipid peroxidation and oxidative stress, which directly reveals the extent of lipid peroxidation and indirectly reflects the extent of cellular damage [45]. SOD is an important enzyme that plays a crucial role in the antioxidant defense system by converting superoxide radicals to hydrogen peroxide and oxygen. GSH-PX and CAT are also critical antioxidant enzymes that help protect cells from oxidative damage by reducing hydrogen peroxide and lipid peroxides. GSH is a tripeptide that is synthesized by the liver, and it is an important antioxidant molecule. Decreased SOD, GSH-PX, CAT, and GSH levels have been observed in patients with alcoholic liver injury, indicating that decreased antioxidant defense mechanisms contribute to the development of liver injury [44,45]. In this study, the levels of ROS, protein carbonyls (PCO), and MDA in the alcohol group were substantially elevated, while the contents of SOD, GSH, GSH-Px, and CAT were significantly reduced. However, the WOPs intervention reversed this change compared to results in the alcohol control group, increasing the content of antioxidant substances and reducing the production of harmful oxidative stress products, suggesting that WOPs have a strong antioxidant capacity and can inhibit excessive oxidative stress and lipid peroxidation, thus exerting the effect of protecting the hepatocytes and improving liver function.

The intestine is the main organ for alcohol absorption, and excessive alcohol intake can cause intestinal bacterial overgrowth, decreased intestinal barrier function, increased permeability, and allow the entry of bacteria and bacterial metabolites such as LPS into the blood circulation, which can cause endotoxemia [46,47]. Endotoxemia leads to an excessive release of inflammatory cytokines, which further aggravates intestinal mucosal barrier damage [18]. DAO is an intracellular enzyme that is mainly found in the cytoplasm of intestinal villous cells, while it is less abundant and less active in other tissues [20]. Once intestinal mucosal cells are damaged and the mucosal barrier is broken, DAO will be released into the blood or will enter the intestinal lumen with the shedding of intestinal mucosal cells, which will, in turn, cause an increase in blood and intestinal luminal DAO activity [20,48]. Since DAO activity is stable in peripheral blood, changes in DAO in peripheral blood are generally detected to reflect intestinal mucosal injury and repair [49]. D-lactate is a metabolite of intestinal flora bacteria, and its levels are closely related to intestinal permeability [20]. In this study, the levels of LPS, DAO, and D-lactate were significantly elevated in the alcohol group, while the intervention of WOPs significantly reduced the elevation of LPS and D-lactate levels, indicating that WOPs have the effect of alleviating alcohol-induced intestinal epithelial cell damage and inhibiting endotoxemia.

Inflammation is one of the main characteristics of ALD, and one of the crucial mechanisms of alcohol-induced liver injury is the direct damage to hepatocytes caused by enteric-derived endotoxemia and elevated levels of inflammatory factors [50]. Endotoxins not only directly damage hepatocytes, but also bind to clusters of differentiation antigen 14 (CD14) and toll-like receptor-4, subsequently activating Kupffer cells, which in turn activate genetic transcription factors for cytokines and inflammatory mediators, such as NF-κB, causing the release of large amounts of inflammatory mediators, such as TNF-α, IL-6, IL-1 [23,29,51,52]. TNF-α mediates a wide range of cellular responses, including inflammation, apoptosis, and fibrosis, which contribute to the development and progression of ALD [23]. Meanwhile, TNF-α is an NF-κB activator, which is mutually stimulated with other inflammatory factors, inducing a cascade amplification response and aggravating liver injury [53]. In this study, serum TNF-α, IL-1β, and IL-6 levels were remarkably elevated in the alcohol group, indicating that alcohol intake caused enteric-derived endotoxemia and produced large numbers of inflammatory factors. After the intervention of WOPs, serum LPS, TNF-α, IL-1β, and IL-6 concentrations were significantly reduced, suggesting that WOPs can ameliorate the liver damage caused by alcohol-induced inflammatory responses. Further detection of related proteins involved in the inflammatory pathway revealed that hepatic NFκB p65 expression levels were remarkably increased in the alcohol group, while NF-κB p65 expression levels were significantly reduced in the high-dose group of WOPs. The results indicated that the WOPs intervention may exert their hepatoprotective effects by inhibiting the expression of NF-κB p65 in the liver and reducing the production of the downstream inflammatory factors such as TNF-α, hopefully providing a new target for WOPs to contribute to the prevention and treatment of ALD.

## 5. Conclusions

In conclusion, this study investigated the protective effects of WOPs—along with their possible mechanisms—on rats with acute alcoholic liver injury. The results showed that WOPs could significantly reduce the serum transaminase level, reduce the degree of liver steatosis, enhance the activity of hepatic ethanol metabolizing enzymes, increase the activity of antioxidant enzymes and antioxidant substances, reduce the level of lipid oxidation products and proinflammatory factors, and inhibit the expression of NF-κBp65 in rats with alcoholic liver injury. Therefore, WOPs exhibit a protective effect against acute ethanol binge-induced liver damage, and the high-dose WOPs (880 mg/kg.bw) exerted the most significant hepatoprotective effect, which is beneficial for the early prevention and treatment of ALD.

## Figures and Tables

**Table 1:** The effect of WOPs on the loss of righting reflex of rats.

Groups	Number	LORR Rate (%)	Latency Time of LORR (min)	Duration Time of LORR (min)
Alcohol	10	80	35.75 ± 32.14	711.25 ± 200.63
Alcohol + WHP (440)	10	80	63.88 ± 40.00	594.37 ± 198.06
Alcohol + WOPs (220)	10	40	78.00 ± 61.17	369.25 ± 149.50 ^##^
Alcohol + WOPs (440)	10	30 ^#Δ^	114.33 ± 50.50 ^#^	331.67 ± 86.05 ^##Δ^
Alcohol + WOPs (880)	10	30 ^#Δ^	128.33 ± 76.00 ^##^	344.64 ± 107.53 ^##Δ^

The rate of LORR was analyzed by Fisher’s exact probability test. Values representing latency time of LORR and duration time of LORR are expressed as the mean ± SD and were analyzed by one-way analysis of variance (ANOVA). ^#^ *p* < 0.05, ^##^ *p* < 0.01, versus alcohol group; ^Δ^ *p* < 0.05 versus whey protein group. LORR, loss of righting reflex; WHP, whey protein; WOPs, walnut oligopeptides.

**Table 2:** Effects of WOPs on serum biochemical parameters of rats.

Parameters	Normal	Alcohol	Alcohol + WHP (440)	Alcohol + WOPs (220)	Alcohol + WOPs (440)	Alcohol + WOPs (880)
ALT (U/L)	52.67 ± 10.03	85.86 ± 21.79 **	71.50 ± 22.05	63.78 ± 9.72 ^#^	59.22 ± 15.43 ^##^	64.17 ± 10.40 ^#^
AST (U/L)	169.17 ± 34.30	264.22 ± 64.26 **	234.78 ± 57.35 *	200.40 ± 50.87 ^#^	195.00 ± 42.99 ^#^	193.56 ± 41.21 ^#^
TC (mmol/L)	1.68 ± 0.22	1.52 ± 0.15	1.60 ± 0.25	1.54 ± 0.28	1.64 ± 0.25	1.42 ± 0.10
TG (mmol/L)	0.67 ± 0.149	1.07 ± 0.16 **	1.05 ± 0.31 **	1.08 ± 0.20 **	0.94 ± 0.25 *	0.80 ± 0.11 ^#Δ^
HDL-C (mmol/L)	0.74 ± 0.10	0.64 ± 0.10	0.75 ± 0.11	0.75 ± 0.16	0.74 ± 0.19	0.70 ± 0.14
LDL-C (mmol/L)	0.27 ± 0.07	0.34 ± 0.07	0.31 ± 0.07	0.28 ± 0.08 *	0.29 ± 0.07	0.24 ± 0.09 ^##^
VLDL (μg/mL)	175.15 ± 18.81	187.32 ± 18.95	180.80 ± 127.32	177.65 ± 28.39	163.07 ± 25.80	160.95 ± 16.09 ^#^
TP (g/L)	71.62 ± 5.85	55.10 ± 5.05 **	58.60 ± 6.50 **	60.21 ± 7.11 **	63.79 ± 7.87 **^##^	63.44 ± 7.47 **^##^
ALB (g/L)	36.33 ± 2.72	30.91 ± 3.52 *	31.46 ± 3.06 *	32.19 ± 2.70 *	33.45 ± 3.93	33.64 ± 2.26
GLB (g/L)	35.28 ± 3.88	25.92 ± 4.41 **	27.14 ± 3.61 **	28.02 ± 4.64 **	30.29 ± 4.50 **^##^	30.67 ± 4.73 **^##^
A:G ratio	1.03 ± 0.06	1.20 ± 0.13 **	1.17 ± 0.07 **	1.16 ± 0.12 **	1.06 ± 0.10 ^##Δ^	1.08 ± 0.11 ^##^
ALP (U/L)	142.17 ± 25.63	278.09 ± 37.95 **	276.75 ± 50.33 **	206.10 ± 32.51 *^##^	205.64 ± 32.50 *^##^	203.33 ± 18.17 *^##Δ^
CR (umol/L)	51.83 ± 7.86	42.40 ± 5.27 *	42.38 ± 5.58 *	43.13 ± 8.74 *	44.00 ± 8.37 *	45.00 ± 5.03
BUN (mmol/L)	5.93 ± 1.59	6.77 ± 1.79	6.40 ± 2.23	5.75 ± 1.96	5.64 ± 1.97	5.55 ± 1.15
TBil (umol/L)	1.68 ± 0.33	0.62 ± 0.64 **	0.54 ± 0.33 **	0.69 ± 0.38 **	0.55 ± 0.40 **	0.71 ± 0.41 **

* *p* < 0.05, ** *p* < 0.01 versus normal control group; ^#^ *p* < 0.05, ^##^ *p* < 0.01 versus alcohol group; ^Δ^ *p* < 0.05 versus whey protein group. ALT, alanine aminotransferase; AST, aspartate aminotransferase; TC, total cholesterol; TG, triglyceride; HDL-C, high-density lipoprotein cholesterol; LDL-C, low-density lipoprotein cholesterol; VLDL, very low-density lipoprotein cholesterol; TP, total protein; ALB, albumin; GLB, globulin; A:G ratio, albumin: globulin; ALP, Alkaline phosphatase; CR, Creatinine; BUN, blood urea nitrogen; TBil, total bilirubin; WHP, whey protein; WOPs, walnut oligopeptides.

**Table 3:** The score results of the pathological changes in the livers of rats.

Group	Number under Each Steatosis Grade					Average Rank
	0	I	II	III	IV	
Normal	6	0	0	0	0	6.42
Alcohol	0	0	1	2	3	30.42 *
Alcohol + WHP (440)	1	1	2	1	1	23.00
Alcohol + WOPs (220)	0	3	2	1	0	17.50
Alcohol + WOPs (440)	1	1	2	2	0	18.92
Alcohol + WOPs (880)	2	2	1	1	0	14.92

The Kruskal–Wallis test was used for the histological examination comparison (*n* = 6 per group). * *p* < 0.05 versus normal control group. WHP, whey protein; WOPs, walnut oligopeptides.

## Data Availability

The data presented in this study are available on request from the corresponding author. The data are not publicly available due to privacy. The studies not involving humans.
